# Translationally controlled tumour protein TCTP is induced early in human colorectal tumours and contributes to the resistance of HCT116 colon cancer cells to 5-FU and oxaliplatin

**DOI:** 10.1186/s12964-017-0164-3

**Published:** 2017-02-01

**Authors:** Ulrich-Axel Bommer, Kara L. Vine, Prianka Puri, Martin Engel, Lisa Belfiore, Karen Fildes, Marijka Batterham, Alistair Lochhead, Morteza Aghmesheh

**Affiliations:** 1 Illawarra Health and Medical Research Institute (IHMRI), University of Wollongong, Northfields Avenue, Wollongong, NSW 2522 Australia; 2 Graduate School of Medicine, University of Wollongong, Northfields Avenue, Wollongong, NSW 2522 Australia; 3 School of Biological Sciences, Faculty of Science, Medicine and Health, University of Wollongong, Wollongong, 2522 NSW Australia; 4 Present address: Southeast Sydney Illawarra Area Health Services, Sydney, NSW Australia; 5 School of Mathematics and Applied Statistics, Faculty of Engineering and Information Sciences University of Wollongong, Wollongong, 2522 NSW Australia; 6 Southern IML Pathology Wollongong, 2500 Wollongong, NSW Australia; 7 Present address: Syd-Path, St. Vincent’s Hospital Darlinghurst, Sydney, 2010 NSW Australia; 8 Illawarra Cancer Care Centre, The Wollongong Hospital, Wollongong, 2500 NSW Australia

**Keywords:** Translationally controlled tumour protein (TCTP), Human colorectal tumours, HCT116 colon cancer cells, 5-fluorouracil (5-FU), Oxaliplatin, PI3-kinase/Akt/mTORC1 signalling pathway, Translational regulation, Chemoresistance

## Abstract

**Background:**

Translationally controlled tumour protein TCTP is an anti-apoptotic protein frequently overexpressed in cancers, where high levels are often associated with poor patient outcome. TCTP may be involved in protecting cancer cells against the cytotoxic action of anti-cancer drugs. Here we study the early increase of TCTP levels in human colorectal cancer (CRC) and the regulation of TCTP expression in HCT116 colon cancer cells, in response to treatment with the anti-cancer drugs 5-FU and oxaliplatin.

**Methods:**

Using immunohistochemistry, we assessed TCTP levels in surgical samples from adenomas and adenocarcinomas of the colon, compared to normal colon tissue. We also studied the regulation of TCTP in HCT116 colon cancer cells in response to 5-FU and oxaliplatin by western blotting. TCTP mRNA levels were assessed by RT-qPCR. We used mTOR kinase inhibitors to demonstrate mTOR-dependent translational regulation of TCTP under these conditions. Employing the Real-Time Cell Analysis (RTCA) System and the MTS assay, we investigated the effect of TCTP-knockdown on the sensitivity of HCT116 cells to the anti-cancer drugs 5-FU and oxaliplatin.

**Results:**

1. TCTP levels are significantly increased in colon adenomas and adenocarcinomas, compared to normal colon tissue. 2. TCTP protein levels are about 4-fold upregulated in HCT116 colon cancer cells, in response to 5-FU and oxaliplatin treatment, whereas TCTP mRNA levels are down regulated. 3. mTOR kinase inhibitors prevented the up-regulation of TCTP protein, indicating that TCTP is translationally regulated through the mTOR complex 1 signalling pathway under these conditions. 4. Using two cellular assay systems, we demonstrated that TCTP-knockdown sensitises HCT116 cells to the cytotoxicity caused by 5-FU and oxaliplatin.

**Conclusions:**

Our results demonstrate that TCTP levels increase significantly in the early stages of CRC development. In colon cancer cells, expression of this protein is largely upregulated during treatment with the DNA-damaging anti-cancer drugs 5-FU and oxaliplatin, as part of the cellular stress response. TCTP may thus contribute to the development of anti-cancer drug resistance. These findings indicate that TCTP might be suitable as a biomarker and that combinatorial treatment using 5-FU/oxaliplatin, together with mTOR kinase inhibitors, could be a route to preventing the development of resistance to these drugs.

**Electronic supplementary material:**

The online version of this article (doi:10.1186/s12964-017-0164-3) contains supplementary material, which is available to authorized users.

## Background

Colorectal cancer (CRC) is a major health problem worldwide; it is the third most common malignancy diagnosed in both men and women, and it affects more than one million people annually [1–3]. Approximately 50% of people diagnosed with CRC will progress to metastatic disease, and the average 5-year survival rate is only 50-60%, resulting in nearly 700,000 deaths worldwide in 2012 [4]. Early detection of the disease onset is hampered by the limited availability of suitable biomarkers. Carcino-embryonic antigen (CEA), still the most widely accepted prognostic marker in CRC, is mainly used for disease monitoring after therapy, since elevated CEA levels are only detected at later stages of the disease [1, 5]. Therefore, there is a need for development of a biomarker that can detect early stage disease and even early growth, such as adenoma.

Chemotherapy is routinely used for patients with stage III and IV colon cancer. Standard chemotherapy for adjuvant treatment of stage III colon cancer is limited to oxaliplatin and 5-fluorouracil (5-FU). The same chemotherapy drugs are indicated for stage IV disease, in addition to irinotecan, or for locally advanced or metastatic disease [6, 7]. There is no other approved chemotherapy agent that can be used in colon cancer with adequate efficacy. Only few biological agents have shown activity in colon cancer during the last few years [8, 9]. Despite these recent advances, there remains an unmet need for overcoming resistance to conventional chemotherapy [10].

Here we study the regulation of translationally controlled tumour protein (TCTP) both early in the development of CRC and in the response of colorectal cancer cells to treatment with oxaliplatin and 5-FU. TCTP (gene symbol: TPT1), also referred to as histamine-releasing factor (HRF) or fortilin, is a highly-conserved, multifunctional protein, which has been implicated in a range of cell biological, as well as disease processes, in particular cancer [11, 12]. One of the best-characterised functions of TCTP is its anti-apoptotic activity [12–15], which is underscored by the demonstration that gene knockout of TCTP in mice is embryonically lethal, due to excessive apoptosis early in embryogenesis [16, 17]. Moreover, TCTP functions as a cytoprotective protein, active in cellular responses to a wide range of stresses, such as oxidative stress [18, 19], heat shock [20], or Ca^++^-stress [21]; it is also involved in the cellular DNA damage repair system [22].

Apart from the cellular stress response, TCTP participates in various other important biological processes, such as protein synthesis [23–25], cell division and early development [11, 12]. TCTP binds to and stabilises microtubules, inclusive of the mitotic spindle [26]. Detachment of TCTP from the spindle through phosphorylation by the mitotic protein kinase Plk-1 is essential for the orderly progression through both mitotic [27] and meiotic [28] cell division.

A substantial body of evidence demonstrates that TCTP is related to cell growth and to the development of cancer [11, 12, 29, 30]. TCTP is overexpressed in many cancer cell lines, and its protein levels are positively related to the growth behaviour of these cells, to their tumorigenicity [11] and even to their metastatic potential [31, 32]. Elevated TCTP levels were detected in a range of human tumours [12], and a high TCTP status has been associated with a poor outcome in breast [31], hepatocellular [32], and in ovarian cancers [33], as well as in gliomas [34]. Work on the experimental ‘tumour reversion model’ has been instrumental in elucidating the importance of TCTP early in cancer development [29, 35, 36]. The anti-apoptotic activity of TCTP has been described as an underlying mechanism, in particular its demonstrated antagonism to the tumour suppressor protein p53 [31, 37, 38]. A more recent report has highlighted the involvement of TCTP in the induction of the epithelial to mesenchymal transition (EMT), a hallmark for the development of cell migration, invasion and metastasis [39]. Consistent with this, TCTP has been suggested as an independent prognostic maker for a poor outcome in breast cancer [31, 40] and in hepatocellular cancer [32].

Several reports even propose TCTP as a potential therapeutic target in breast [31, 40] and prostate cancer [11, 41], and in neurofibromatosis type 1-associated tumours [42]. Successful targeting of TCTP with antisense-oligonucleotides has been demonstrated in castration-resistant prostate cancer in mice [41]. Much earlier, TCTP was described as a target of the antimalarial drug artemisinin in *Plasmodium* [43]. Dihydro-artemisinin (DHA) has also anti-cancer activity, and it targets TCTP in cancer cells [40, 44–46], resulting in proteasome-mediated degradation and reduction in cellular TCTP levels [40, 45]. In an early study on the ‘tumour reversion model’, several anti-histaminic drugs, as well as some anti-depressants, were found to be active in reducing TCTP levels in cancer cells [36]. This led to the search for further anti-histaminics as TCTP inhibitors with antiproliferative activity [47] and to more detailed mechanistic studies that underlie the anti-cancer activity of these antidepressant drugs [29, 31].

Up until recently, relatively little was known about the role and regulation of TCTP in colorectal cancer. In former reports, Northern blot analysis was used to demonstrate overexpression of TCTP mRNA in colon carcinoma cell lines [48]. In a microarray analysis study, TCTP mRNA was found to be up-regulated in primary tumours from colon cancer patients with lymph node metastases [49]. Two papers reported that TCTP-knockdown inhibited proliferation, invasion and metastatic potential of LoVo colon cancer cells [50, 51], indicating that TCTP is indeed involved in colon cancer progression.

Recently, we demonstrated that growth factor-dependent expression of TCTP is translationally regulated in both HeLa and HT29 colon cancer cells through the PI3-K/Akt/mTORC1 signalling pathway [52]. Since this pathway is frequently upregulated in CRC [53–55], we hypothesised that overexpression of TCTP in CRC is driven by this pathway as well. First, we asked whether the overexpression of TCTP occurs early in the development of CRC. We used immunohistochemistry to assess TCTP protein levels in panels of surgical CRC samples from adenomas, and adenocarcinomas, compared to surrounding normal colon tissue. We conclude from our results that, TCTP levels are elevated early in cancer development. To study the regulation of TCTP in colorectal cancer cells under controlled conditions, we chose the HCT116 colon cancer cell line. We asked whether TCTP is regulated in these cells under conditions of treatment with two drugs commonly used in CRC therapy, 5-FU and oxaliplatin. TCTP protein levels are significantly upregulated in these cells by treatment with either of these two drugs, and this regulation is mediated through the mTORC1 signalling pathway. Knockdown of TCTP sensitises HCT116 cells to the treatment with 5-FU or oxaliplatin, which indicates that TCTP up-regulation is part of the stress response of colorectal cancer cells to the treatment with these DNA-damaging anti-cancer drugs. Increased TCTP levels are therefore likely to contribute to the development of chemoresistance to these drugs, as it frequently occurs in CRC.

## Methods

### Patient samples

To assess TCTP expression levels in paraffin-embedded surgical samples of colorectal cancer by immunohistochemistry, we performed a database search in the Pathology records from the Illawarra Cancer Care Centre of The Wollongong Hospital, and from Southern.IML Pathology, Wollongong. Based on these records, about 70 patients with a colorectal pathology were selected, with the aim to obtain an even spread of the following diagnoses: adenomas, adenocarcinomas (non-metastatic), and adenocarcinomas (metastatic; lymph nodes only). Normal colon tissue was typically identified in the surrounding, non-malignant tissue of the tumour samples. Ethics approval for the project was obtained from the Human Ethics Committee of the University of Wollongong. The formalin-fixed, paraffin-embedded patient samples were sourced from the respective Pathology laboratories, freshly sliced and subjected to immunohistochemistry. Post-staining, samples were de-identified, numbered and grouped according to the three pathology types mentioned above.

### Immunohistochemistry

Immunoperoxidase staining of TCTP expression was performed on formalin-fixed, paraffin-embedded sections of the patient samples using the BondmaX automated processing module (Leica Biosystems). Dewax and retrieval was performed on board the instrument. Epitope retrieval was done at low pH for 30 min. Sections were incubated with the TCTP antibody at room temperature for 1 h (rabbit polyclonal antibody to TCTP - AB37506; 1:000). Hydrogen peroxide was applied after primary antibody to quench endogenous peroxidase. Detection of the primary antibody was performed with anti-rabbit poly-HRP-IgG, using DAB as substrate. Samples were counterstained with HE. The slides were microscopically inspected and scored independently by a medical student and two trained Pathologists. Scoring was performed according to staining intensity (score 0 – negative, score 1 – weak, score 2 – strong) and according to numbers of cells stained (score 0: 0-5% of cells; score 1: 5–75% of cells; score 2: >75% of cells). The two numerical scores from intensity and cell percentage were multiplied to give a final score of 0, 1, 2, or 4. Total score was compared between groups using a Kruskal-Wallis test with post-hoc analysis adjusted for multiple comparisons. The analysis was performed using SPSS V21 (IBM SPSS, IBM Corporation, Armonk NY, USA).

### Cell lines and culture conditions

The human colorectal carcinoma cell line (HCT116) was purchased from the American Type Culture Collection (ATCC, USA) and routinely cultured in RPMI-1640 medium (Invitrogen, USA) containing 10% (v/v) foetal calf serum (FCS; Thermo, USA). When 80% confluence was reached, cells were detached by incubation with 5 mM trypsin/EDTA and harvested after centrifugation in a Heraeus Megafuge 1.0 (Thermo Scientific, USA) at 1200 rpm for 5 min at room temperature. Cells were resuspended in media and viable cells counted using a haemocytometer and trypan blue staining. All cell lines were confirmed negative for mycoplasma contamination.

### Western blot analyses

For preparation of extracts, HCT116 cells were lysed in NP40 cell lysis buffer (Life Technologies, Burwood, VIC Australia) with freshly added 1 mM DTT and protease inhibitor cocktail (Sigma-Aldrich #P8340). Lysates were centrifuged for 10 min at 15,000 rpm and 4 °C. Supernatants were stored at −80 °C and protein concentrations were determined using the Bradford assay. Equal amounts of cell extract (typically 15 μg total protein) were separated by SDS-gel electrophoresis on 12.5% polyacrylamide gels (Criterion XT precast gels; Bio-Rad, Gladesville, NSW Australia), and electrophoretically transferred onto polyvinylidene difluoride (PVDF) membranes. Blots were blocked in 3% (w/v) fat-free milk and incubated with the indicated primary antibody overnight at 4 °C. For visualisation, blots were incubated with HRP-linked secondary antibodies, developed with Lumi-Glo (Cell Signalling Technology; Genesearch, Arundel, QLD Australia) and exposed to Kodak X-Omat film. After scanning (GS-800 scanner; Bio-Rad, Gladesville, NSW Australia), signals were quantified using the Multi-Analyst software (Bio-Rad).

### Antibodies

Anti-TCTP antibodies AB37506 (polyclonal; for immunohistochemistry), and AB58362 (monoclonal; for western blotting) were purchased from Abcam (UK) via Sapphire Bioscience, Waterloo, NSW Australia. Phospho-ribosomal protein S6 (Ser 240/244; #2215), and secondary HRP-linked antibodies were from Cell Signalling Technology, purchased via Genesearch, Arundel, QLD Australia. Anti-α-and β-tubulin antibodies were from Amersham Biosciences (UK).

### Drugs and inhibitors

Anti-cancer drugs 5-fluorouracil (5-FU) and oxaliplatin were purchased from Sigma-Aldrich. The drugs were kept as 10 mM stock solutions in sterile PBS at −20 °C. The mTOR kinase inhibitors AZD8055 and PP242 were obtained from Selleck via Life Research, Scoresby, Victoria Australia. The inhibitors were kept as 10 mM stock solutions in DMSO at −80 °C.

### Quantitation of TCTP mRNA in HCT116 cells by reverse transcription qPCR

HCT116 cell cultures were treated with 5-FU (20 μM) or oxaliplatin (12.5 μM) for 48 h (*n =* 4/group). After treatment, cultures were processed for RNA extraction with Tri-reagent (TR 118, Medical Research Centre) according to the manufacturer’s instructions. Genomic DNA was removed using the Ambion Turbo DNA-free kit, followed by reverse transcription using an M-MLV reverse transcriptase for 2 h at 37 °C (M1705, Promega). Quantitative real time PCR was carried out in technical triplicates on a Light Cycler 480 (Roche) using the SYBR select master mix (4472918, Life Technologies) according to manufacturer’s instructions. Reactions for each target were optimised for an efficiency >1.8 and specificity was confirmed via melt-curve analysis. The following primers (KiCqStart SYBR green, Sigma) were used: translationally controlled tumour protein (TCTP/TPT1) (sense, 5’-TACTCTTTCTGGTCTCTGTTC-3’; antisense, 5’-CAAGTTTCACAAAAGAAGCC-3’), and β2-tubulin (B2T) (sense, 5’-AAGGACTGGTCTTTCTATCTC-3’; antisense, 5’-GATCCCACTTAACTATCTTGG-3’) at 400 nM in 20 μL reactions with 80 ng cDNA. Treatment effects on TCTP mRNA expression were assessed using one-way ANOVA, followed by Tukeys post-hoc analysis, where applicable. Analyses were performed using SPSS 19.0 (IBM). Statistical significance was accepted at *P <* 0.05 and data are presented as the mean ± standard error of the mean (SEM).

### TCTP-knockdown

To knock down TCTP expression, HCT116 cells were transfected with the TPT1-3 siRNA (Ambion) initially, and more recently with the Silencer SELECT siRNA (TPT1 Gene; siRNA # s14360) (Ambion; purchased from Life Technologies Australia) using Lipofectamine™ RNAiMAX as transfection reagent (LifeTechnologies), according to the reverse transfection protocol for HCT116 cells provided by the manufacturer (Invitrogen). The knockdown efficiency was assessed by western blotting from transfection assays in 24-well format; at an siRNA concentration of 15 nM (48 h), we observed a knock-down to 6-9% of control TCTP levels. For proliferation assays and real-time cell analysis assays, the test format was adjusted to the 96-well plate size. In our conditions, we noted some off-target effects by the commercially available control siRNAs; therefore, we moved to using siRNA for Luciferase, an unrelated gene not present in mammals, as a control siRNA, or using GAPDH-siRNA in some experiments.

### Real-time cell analysis

The effect of increasing concentrations of 5-FU or oxaliplatin on cell proliferation was determined using the xCELLigence RTCA System (Roche Applied Science) in specialised 16-well E-16 plates. Transfection mixes containing Lipofectamine™ RNAiMAX and either TCTP-siRNA, or Luc-siRNA (in some experiments GAPDH-siRNA) as a control, were made up in Opti-MEM medium (Life Technologies). Following the reverse transfection method for TCTP knock-down experiments, 40 μL of the transfection mix (or plain Opti-MEM medium for controls) were added to the wells of the E-16 plates first. Then HCT116 cells were added at 5000 cells/well in a volume of 140 μL RPMI medium. The cells were allowed to settle for 30 min at room temperature before being incubated at 37 °C for 24 h. Varying concentrations of 5-FU or oxaliplatin (in 20 μL sterile PBS, pH 7.4) were then added to give a total volume of 200 μL and the plate then incubated at 37 °C for further 72 h. Comparisons were made to untreated cells, which received 20 μL of sterile PBS. The viability and proliferation of cells was measured as a dimensionless parameter termed cell index (CI) in real-time. CI is derived from the relative change in measured electrical impedance, whereby electrical impedance represents the presence and spread of adherent cells. Real-time data was accumulated every 60 min and graphed using the RTCA-DP Software (version 1.2.1), supplied with the xCELLigence System.

### Cell proliferation assay

The Cell Titre 96® AQueous One Solution Cell Proliferation Assay (MTS assay, Promega) was used to evaluate the viability of cells after treatment with the DNA damaging agents 5-FU or oxaliplatin as described by Vine et al. [56]. Cells (2–3 × 10^3^ cells/well) were seeded in 96-well plates in a total volume of 180 μL and incubated at 37 °C, 5% CO_2_, for 24 h. 5-FU or oxaliplatin (1–100 μM) were added to the cells (in 20 μL) and incubated for 72 h. MTS reagent was added and allowed to incubate for 3 h before the absorbance was measured at 490 nm using a microplate reader (Spectramax, Molecular Devices, USA). IC_50_ values (the dose required to inhibit the metabolic activity of 50% of the cell population) were calculated from logarithmic sigmoidal dose response curves generated using GraphPad Prism v6 software (GraphPad Inc). Data is presented as a mean ± standard deviation (SD) from ≥ 2 independent experiments, performed in triplicate.

## Results

### TCTP is induced early in the development of human colorectal tumours

**Fig. 1:**
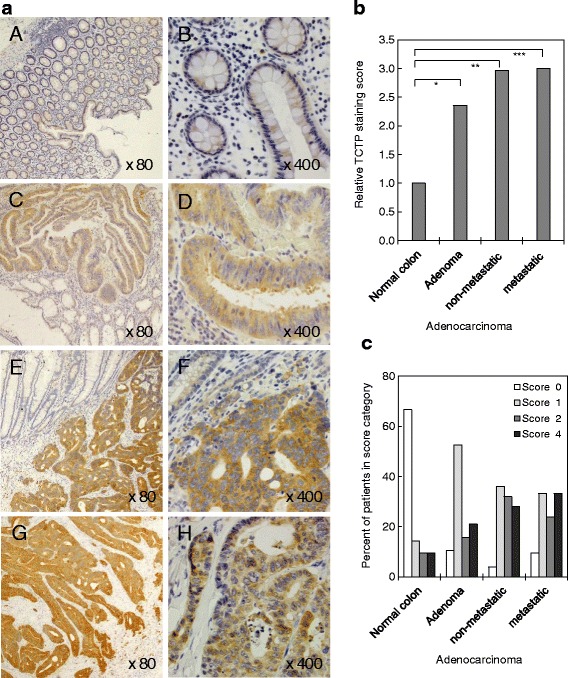
TCTP protein levels increase significantly in the early stages of human colon cancer development. **a** Immunohistochemical staining with an anti-TCTP antibody of paraffin-embedded tumour samples from colon cancer patients with confirmed adenomas (*C *, *D*), non-metastatic (*E*, *F*) or metastatic adenocarcinomas (*G*, *H*). Areas from surrounding normal colon were used as a control (*A*, *B*). Representative examples are shown for each group at 80 × and 400 × magnification. **b** Graph showing the average TCTP scoring for each patient group, normalised to TCTP levels in normal colon tissue (*P = 0.052; **P = 0.001; ***P < 0.001). **c** Graph representing the score distribution for each patient group (see Methods for scoring)

In order to detect alterations of TCTP protein levels early during development of colorectal cancer, we performed immunohistochemistry for human TCTP on paraffin-embedded surgical samples from about 70 patients. The patient samples were grouped into three groups (about 22 patients each), according to the following diagnoses: adenomas, adenocarcinomas (non-metastatic), and adenocarcinomas (metastatic; lymph nodes only). For comparison, normal colon tissue was identified during microscopic inspection in the surrounding, non-malignant tissue of the tumour samples. A panel of example images from slides of the three tumour groups, compared to normal tissue, is shown in Fig 1a. Images C, E and F also provide a direct comparison between cancerous and adjacent normal tissue.

These images demonstrate that TCTP protein levels are clearly higher in the tumour samples, compared to normal colon tissue. To quantify the results, the staining intensity as well as numbers of cells stained were scored for each sample as described in the Methods section. The resulting score figures were compared between groups and plotted for the three CRC tumour groups, compared to the normal colon tissue in Fig 1b. Figure 1c represents the relative score distribution within each tissue group, indicating that higher score numbers are typically more represented in adenocarcinomas, compared to adenomas and normal tissue. The results in Fig. 1b clearly demonstrate a significant increase in TCTP levels (up to about 3-fold) in the development of colorectal cancer, during transition from normal tissue to the adenoma (*P =* 0.052) and the adenocarcinoma stage (*P =* 0.001 for the non-metastatic and *P <* 0.001 for the metastatic group). The observation that the investigated tumour samples display significantly elevated TCTP levels, compared to normal colon tissue, already at the adenoma stage would indicate the TCTP induction occurs early during colon cancer development, whereas no changes were observed during later stages (cf. non-metastatic vs. metastatic adenocarcinomas, *P =* 1.000).

We also asked whether there is a correlation of the TCTP score with other clinic-pathological parameters. However, we were unable to detect any such correlation with BMI, age, gender, or measured CEA levels. This was largely because some of the clinical data were incomplete and thus the overall sample size was too small to adequately power the detection of these differences.

### TCTP levels are increased in HCT116 colon cancer cells in response to 5-FU and oxaliplatin

**Fig. 2:**
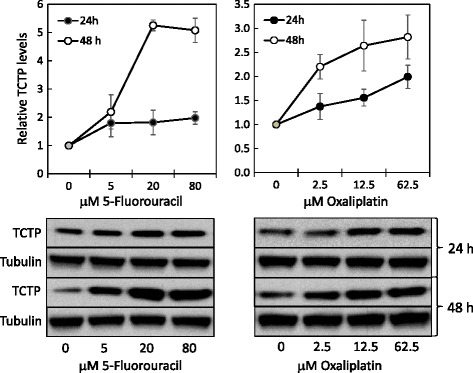
Treatment of HCT116 colon cancer cells with 5-FU and oxaliplatin results in increased TCTP protein levels. Cells were seeded in 12-well plates, grown to about 70% confluency and treated with either 5-FU or oxaliplatin at the indicated concentrations for 24 or 48 h. TCTP levels were assessed by western blotting. The bottom panels show representative western blots for TCTP and α-tubulin as a loading control. TCTP signals were quantified and normalised for the corresponding α-tubulin signals. In the graphs, normalised TCTP levels are plotted against the drug concentration for both the 24 h (filled symbols) and the 48 h treatment time (open symbols). Dots represent the average of three experiments ± SD

In order to understand how TCTP levels are regulated in colorectal cancer, we have chosen to study TCTP regulation in the colon cancer cell line HCT116 under conditions when cells are subjected to stress induced by DNA-damaging anti-cancer drugs. TCTP is known to be a cytoprotective protein that protects e.g. breast cancer cells from oxidative stress [18] and that is also involved in the DNA-damage response [22]. We therefore anticipated that colon cancer cells would respond to treatment with such anti-cancer drugs by alterations in the expression of cytoprotective proteins, such as TCTP, as part of the cellular stress response. To test this hypothesis for TCTP, we have chosen 5-fluorouracil (5-FU) and oxaliplatin, two DNA-damaging agents most frequently used in CRC chemotherapy. Treatment of HCT116 cells with increasing concentrations 5-FU or oxaliplatin for 24 h or 48 h did indeed result in a significant increase in endogenous TCTP protein levels (Fig. 2), with the most prominent increase occurring after a 48 h treatment of HCT116 cells with the higher concentrations of 5-FU. A similar, but less pronounced effect was also occurring with oxaliplatin.

### Induction of TCTP expression in HCT116 colon cancer cells in response to 5-FU and oxaliplatin is translationally regulated through the mTORC1 signalling pathway

**Fig. 3:**
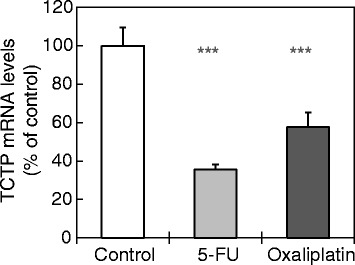
Treatment of HCT116 cells with 5-FU or oxaliplatin results in decreased TCTP mRNA levels. HCT116 cells were treated with 5-FU (20 μM) or oxaliplatin (12.5 μM) or culture medium (control) for 48 h (*n =* 4). TCTP mRNA levels were assessed by RT-qPCR and normalised against β2-tubulin mRNA as reference. Values are presented as mean percentage expression change, compared to untreated control cells ± SEM (***P < 0.001 vs. control)

As for other cytoprotective proteins, TCTP levels can be translationally regulated as part of a swift response, e.g. to cellular stresses. In order to learn whether the observed upregulation of TCTP in HCT116 colon cancer cells is due to transcriptional vs. translational regulation, we assessed the TCTP mRNA levels in HCT116 cells under these conditions, using RT-qPCR. The mRNA of β2-tubulin was used as a reference mRNA. The results plotted in Fig. 3 show that in fact TCTP mRNA levels are not increased, but rather down-regulated through treatment of HCT cells with 5-FU or oxaliplatin. This effect appears to be more pronounced in the case of 5-FU treatment (ca 60% reduction), compared to oxaliplatin (ca. 40%, *P <* 0.001 vs. 5-FU). This result shows that transcriptional regulation is not involved in the observed increase in TCTP protein levels induced by 5-FU or oxaliplatin (Fig. 2).

**Fig. 4:**
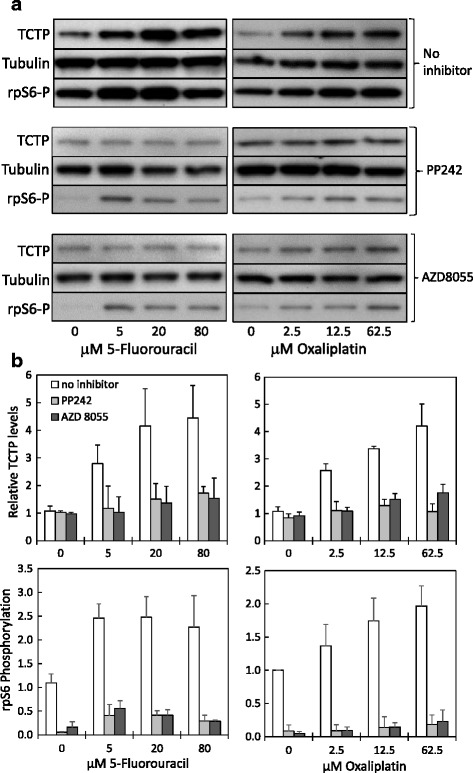
Induction of TCTP expression through 5-FU or oxaliplatin is inhibited by mTOR kinase inhibitors. HCT116 cells were treated for 48 h with 5-FU or oxaliplatin at the indicated concentrations. Where indicated, AZD 8055 (100 nM) or PP242 (1 μM) were added. TCTP levels were assessed by western blotting; signals were quantified and normalised against α-tubulin as a loading control. As a readout of mTOR activity, phosphorylation of the ribosomal protein S6 at Ser 240/244 was assessed using a phospho-specific antibody. **a** Representative western blots demonstrating the alteration of the levels of TCTP protein and of phospho-ribosomal protein S6, respectively, in relation to the drug concentration. **b** Graphs, showing the alteration of TCTP levels (top graphs) and relative rpS6 phosphorylation levels (bottom graphs) against the drug concentration, both in the absence (open bars) or in the presence of the mTOR kinase inhibitors AZD8055 (*dark grey bars*) or PP242 (*light grey bars*). Bars represent the average of three experiments ± SD

We have recently shown that TCTP mRNA translation is regulated through the PI3-K/Akt/mTORC1 pathway [52]. In order to investigate whether the mTORC1 pathway might also be involved in the increase in TCTP protein levels observed here, we employed two mTOR-kinase inhibitors, AZD8055 and PP242, which inhibited TCTP mRNA translation more efficiently than the classic mTOR inhibitor rapamycin [52]. The results shown in Fig. 4 demonstrate that the increase in TCTP protein levels induced by 5-FU or oxaliplatin is completely inhibited by the two mTOR kinase inhibitors. In these western blots, we also probed for the level of phosphorylation of the ribosomal protein S6 (rpS6) at Ser 240/244, which serves as a readout of mTORC1 activity, since mTOR complex 1 activates the p70-S6 kinase, which in turn phosphorylates rpS6 at these sites [57]. Monitoring rpS6 phosphorylation levels confirms that, in the presence of AZD8055 or PP242, mTORC1 is indeed inhibited in our samples. These results also show that, as for TCTP expression levels, mTORC1 activity is enhanced with increasing 5-FU or oxaliplatin concentrations, albeit to a lesser extent (Fig.4a & b). We conclude from these results that the elevation of TCTP levels observed in HCT116 colon cancer cells after treatment of with 5-FU or oxaliplatin is translationally regulated through increased signalling via the mTORC1 pathway.

### TCTP partially protects HCT116 colon cancer cells against the cytotoxic effects of 5-FU and oxaliplatin

**Fig. 5:**
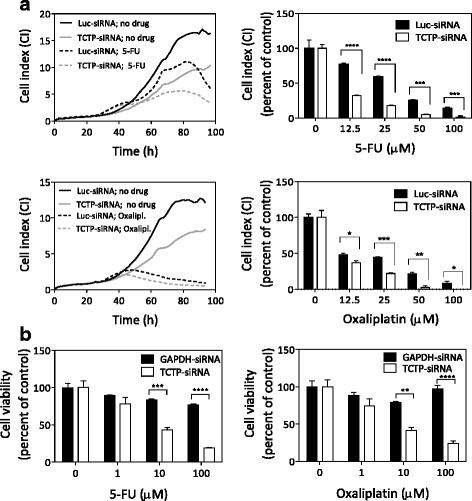
TCTP partially protects HCT116 colon cancer cells against toxicity induced by 5-FU or oxaliplatin. **a** Effect of TCTP knock-down on cellular sensitivity to 5-FU or oxaliplatin, assessed by the the xCELLigence RTCA System. HCT116 cells were either mock-transfected or transfected with TCTP siRNA or Luciferase siRNA (as a control) using Lipofectamine™ RNAiMAX transfection reagent for 24 h, and subsequently incubated in the presence of the indicated concentrations of either 5-FU or oxaliplatin. Cell growth was monitored in real-time using the xCELLigence RTCA System. Representative growth curves are shown in the left panels for control cells and for treatment with 12.5 μM 5-FU or oxaliplatin, as an example. Relative cell numbers are expressed as Cell Index (CI). Right panels compare the relative CI-values for Luc-siRNA, and TCTP-siRNA at the time point, when the control cells reached a CI value of 10 (5-FU treatment) or of 5 (oxaliplatin treatment). **b** Effect of TCTP knock-down on cellular sensitivity to 5-FU or oxaliplatin, measured by the MTS endpoint assay for cytotoxicity. TCTP-siRNA or GAPDH-siRNA (as a control) were transfected into HCT116 cells using Lipofectamine™ RNAiMAX transfection reagent and, after 24 h, incubated in the presence of the indicated concentrations of drugs for another 48 h. Cell viability was assessed using the MTS endpoint assay and was plotted against the concentration of 5-FU (left graph) and that of oxaliplatin (right graph). Statistical significance was ascertained using 2WAY ANOVA (****P < 0.0001, ***P < 0.001, **P < 0.01, *P < 0.05)

To investigate the involvement of TCTP in the protection of colon cancer cells against DNA damaging anti-cancer drugs, we performed TCTP knockdown on HCT116 colon cancer cells and monitored the sensitivity of these cells to 5-FU and oxaliplatin using the xCELLigence Real-Time Cell Analysis (RTCA) System. This system monitors cell growth in real-time by impedance measurement, and results are recorded as Cell Index (CI). In Fig. 5a, we show two graphs with representative growth-curve comparisons for 5-FU- or oxaliplatin-treatment of cells transfected with either TCTP siRNA or luciferase siRNA as a control. The right panels demonstrate quantitative comparisons from selected time points for all drug concentrations and both transfections. The results show that HCT116 cells transfected with TCTP-siRNA were more sensitive to 5-FU or oxaliplatin (over a wide concentration range), compared to HCT116 cells transfected with the non-targeting Luciferase-siRNA. We conclude from these results that TCTP is indeed able to partially protect HCT116 cells against the cytotoxic effects of 5-FU or oxaliplatin, which confirms our hypothesis that the observed induction of TCTP synthesis may be part of the cellular defence in response to the drug treatment.

The RTCA data was then validated using the MTS quantitative colorimetric cell proliferation assay (Fig. 5b). Knockdown of TCTP in HCT116 cells resulted in a significant decrease in cell viability, 2- and 4-fold after treatment with 10 μM and 100 μM 5-FU and oxaliplatin, compared to GAPDH-siRNA transfected HCT116 control cells. The cytoprotective effect of TCTP was much reduced at the lowest drug concentration (1 μM) (Fig. 5b). Overall, the results obtained with the MTS assay validate our conclusion drawn from the data presented in Fig. 5a that TCTP protects HCT116 cells against the cytotoxicity exerted by 5-FU and oxaliplatin.

We also studied the protective effect of TCTP in overexpression experiments. To this end, we utilised a model cell line that stably overexpresses TCTP, which we had originally established for a different project [26]. These are bovine mammary epithelial cells, which harbour either a TCTP-overexpressing plasmid or the empty vector as a control. We used this pair of cell lines, as model mammalian cells, to demonstrate the protective effect of TCTP against the cytotoxic action of 5-FU and oxaliplatin through overexpression, as shown in Additional file 1. In the RTCA assay, TCTP overexpression displays a clear protective effect against the 5-FU- and oxaliplatin-induced cytotoxicity, at least at the higher drug concentrations (left panels). The results of the MTS assay (right panels) show the same trend throughout, although the differences between control cells and TCTP overexpressing cells are much smaller in this case. The reduced effect observed in the overexpression experiment, compared to TCTP knock-down (Fig. 5), can be explained by the fact that the level of overexpression achieved in these cells is only about 2-fold, since higher levels of TCTP are not tolerated by these cells [26]. Overall, our results obtained from both TCTP-knockdown on HCT116 colon cancer cells and the TCTP overexpressing model cell line consistently demonstrate the TCTP is indeed able to protect mammalian cells, and specifically human colon cancer cells, against the cytotoxic effects of the DNA-damaging anti-cancer drugs 5-FU and oxaliplatin.

## Discussion

### TCTP levels in colon cancer

Enhanced expression of TCTP in colorectal cancer has been reported previously. However, earlier studies used colon cancer cell lines, rather than human tumour tissue [48, 50, 51], or they assessed TCTP mRNA levels via Northern blotting [48] or via microarray analysis [49], which does not necessarily reflect alterations in the expression levels of translationally regulated proteins. Two studies reporting an increase of TCTP levels in human colorectal tumours were based on very small sample sizes of 20 [49], or six patients [58], respectively. Only the latter study actually compared protein levels (using proteomics methods), between normal and CRC samples. In order to consolidate these initial observations and to better understand the regulation of TCTP expression in early colon cancer, we undertook this study, using immunohistochemistry on 70 surgical samples from human colorectal tumours. Our results demonstrate that TCTP levels are upregulated in the early stage of colon cancer development, i.e. in adenomas, and they reach their maximum levels already at the stage of non-metastatic adenocarcinoma (Fig. 1). Due to incomplete availability of clinical data, we were unable to establish a more precise correlation to tumour staging, or to other clinical parameters (see results). During the preparation of this manuscript, a new study was published, describing similar observations on a group of 134 colon cancer patients from a Chinese population [59]. These authors also observed a significant increase in TCTP levels already at the adenoma stage, and they established an association of high TCTP levels with high pathological grades, TNM stage IV and poor patient survival. They also found that TCTP levels are higher in metastatic tumour samples compared to primary tumour sites. On the other hand, colon adenomas displayed TCTP levels comparable to those of metastases [59]. Taken together, the results of the Chinese and our study indicate that it would be worthwhile to explore the suitability of TCTP as a biomarker in the early stages of colon cancer development.

At this point, we are not certain about the mechanism underlying the early increase in TCTP levels in CRC. However, we have recently demonstrated that, during growth-factor dependent induction of HeLa cells, and of HT29 colon cancer cells, TCTP levels are up-regulated by signalling through the PI3-K/Akt/mTORC1 pathway [52]. Since others have shown that the mTOR pathway is activated early in the development of CRC [60], it is very likely that activation of this signalling pathway is indeed involved in driving the early increase in TCTP protein levels.

### Regulation of TCTP in colon cancer cells in response to DNA-damaging anti-cancer drugs

Stress-dependent alteration of cellular TCTP levels has been described for a range of conditions (reviewed in [11, 12]); for example in cells exposed to heavy metals [61, 62], to heat shock [20] and to oxidative stress [19, 63]. In particular, TCTP regulation under oxidative stress conditions has been studied in cancer cells, where TCTP was found to partially protect cells against mild oxidative stress [14]. The recent finding that TCTP is also involved in the cellular DNA damage sensing and repair system [22], prompted us to investigate the regulation of TCTP levels in colon cancer cells, in response to two anti-cancer drugs that act as DNA-damaging agents, albeit through different mechanisms. These two drugs, 5-FU and oxaliplatin, are the cornerstone drugs used in chemotherapy of CRC. Treatment of HCT116 colon cancer cells with these drugs resulted in a dose-dependent increase on TCTP levels, which was more pronounced after 48 h, compared to 24 h (Fig. 2). This up-regulation of TCTP is likely to be part of the cellular stress response to the cytotoxicity caused by these drugs.

Elevation of TCTP levels in response to oxaliplatin was reported previously in a proteomics study on three other colon cancer cell lines (HT29, SW260 and LoVo cells), where TCTP was found to be one of 21 proteins upregulated in all three lines [64]. Compared to our results (Fig. 2), in this study the highest TCTP protein levels were observed already at the 24 h time point of treatment. These findings are consistent with an earlier paper reporting increased TCTP levels in melanoma cell lines that are resistant to a range of other anti-cancer drugs, inclusive of etoposide [65]. In contrast, a later investigation on gene expression patterns in 5-FU-resistant vs. normal HCT116 cells failed to detect alterations in TCTP expression in response to 5-FU treatment [66]. However, this study was based on microarray analysis comparing relative mRNA expression, which is unlikely to detect alterations in the expression of genes that are regulated at the translational level. It is also interesting to note that chemical compounds, which exert their anti-proliferative effects through different mechanisms, lead to down-regulation of TCTP expression levels in HCT116 cells [67]. Similarly, antihistaminic [47] or antidepressant drugs [29] are targeting TCTP and were therefore discussed as potential options for anti-cancer treatment.

### Mechanism of TCTP regulation in response to DNA-damaging anti-cancer drugs

TCTP may be regulated at the transcriptional or at the translational level of gene expression, depending on the type of stress condition applied (reviewed in [11, 12]). In order to distinguish, which level of regulation applies to the observed anti-cancer drug dependent increase in TCTP protein levels (Fig. 2), we assessed the alteration of TCTP mRNA levels in HCT116 colon cancer cells, in response to treatment with 5-FU or oxaliplatin. The results show that TCTP mRNA levels are down- rather than up-regulated under these conditions (Fig. 3). This finding indicates that the TCTP mRNA is either transcriptionally downregulated or partly destabilised under the influence of DNA-damaging anti-cancer drugs; it also excludes the possibility that transcriptional regulation is involved in the observed increase in TCTP protein levels. We did not further investigate the mechanism that underlies the observed down-regulation of TCTP mRNA. However, we can exclude a potential artefact due to differential regulation of different mRNA isoforms, since the two known species of TCTP mRNAs differ from one another only by an extension at the 3’-end of the longer mRNA [68]; thus both isoforms are detected by our PCR primers.

It was surprising to find that, despite of lowered mRNA levels (Fig. 3), TCTP protein levels are clearly upregulated in HCT116 colon cancer cells under conditions of treatment with 5-FU and oxaliplatin (Fig. 2). This could be explained either by a powerful upregulation of mRNA translation, which overrides the effect of lowered mRNA levels, or by protein stabilisation. TCTP mRNA belongs to the class of 5’-TOP mRNAs (containing a 5’-terminal oligopyrimidine tract, 5’-TOP) [69], whose translational activity is largely controlled through the mTOR pathway [70]. Consistent with this, during growth-factor dependent up-regulation of TCTP in cancer cells, TCTP mRNA translation is regulated through the PI3-K/Akt/mTORC1 pathway [52]. Therefore, we asked whether the observed increase in TCTP protein levels in response to 5-FU or oxaliplatin treatment (Fig. 2) is also regulated through mTOR. Using two mTOR-kinase inhibitors, we showed that these nearly completely inhibited this increase in TCTP levels (Fig. 4). This result indicates that TCTP protein expression is indeed regulated through the mTORC1 pathway, and it provides additional evidence for translational regulation of TCTP expression under these conditions.

Our observation is contrasting to the well documented believe that cellular stresses, such as hypoxia or genotoxic stress (DNA damage), typically result in an inhibition of mTORC1 activity [71]. Whilst inactivation of mTORC1, and consequently shut down of protein synthesis under cell stress conditions, is generally a sensible biological response, there are also instances, which should allow for exceptions. One of the early papers establishing the link between DNA damage and mTOR regulation observed that initially mTOR inhibition is still reversible; it becomes irreversible only after entry of cells into apoptosis [72]. Also, a distinction has to be made between high and low levels of stress. For example, PTEN, the negative regulator of the PI3-K/Akt/mTORC1 pathway, was found to be suppressed in the colon cancer cell lines HT29 and LoVo at low concentrations of 5-FU and oxaliplatin, allowing for activation of the pathway [73]. This is reminiscent of the regulation reported for TCTP under oxidative stress in breast cancer cells, where mild stress resulted in increased TCTP levels, whereas harsh stress conditions led to reduced TCTP levels [18].

TCTP is in the same league with other, well-known anti-apoptotic proteins, in that these proteins all have a cytoprotective function, they are involved in cancer and they are translationally regulated through the mTORC1 pathway. Examples are the proteins Bcl-2 and Bcl-XL [74], Mcl-1 [75, 76], and survivin, which was shown to be important for promoting motility and metastasis in CRC via translational control mechanisms [77]. Both, Bcl-XL and Mcl-1 have been identified as interaction partners of TCTP (reviewed in [11, 12, 14]), and they have been shown to contribute to enhanced resistance of colorectal cancer cells against 5-FU and oxaliplatin [78]. Recently, a mechanism has been described, by which mTORC1 may be regulated by Bcl-2 and Bcl-XL, i.e. via competition for binding to the mTORC1 inhibiting protein FKBP38 [79]. This is interesting for two reasons: 1. TCTP itself has been implicated in the regulation of mTORC1 in Drosophila, through interaction with Rheb [80], although other studies found different results in mammalian cells [81, 82]. 2. Enhanced Bcl-XL could locally activate mTORC1 [79], and this could explain the observed mTOR-dependent up-regulation of TCTP in the presence of 5-FU. Certainly, there are mechanisms in place to maintain (or even enhance) the synthesis of cytoprotective proteins under stress conditions, when general protein synthesis is shut down [71], however this needs to be shown for each protein individually.

Previous studies aimed at targeting TCTP in cancer cells have identified three groups of established drugs that are able to reduce intracellular TCTP levels, i.e. the antimalarial drug artemisinin [40, 45, 46], anti-histaminics [36, 47] and certain anti-depressants [29, 36]. Of the latter, sertraline, a selective serotonin reuptake inhibitor (SSRI), is of particular interest in the context of colorectal cancer. The potential anti-cancer activity of sertraline was demonstrated in human colon cancer cell lines and in CRC xenografts in mice [83], and two epidemiologic studies, in Canada [84] and the US [85], showed that the use of SSRIs reduces the risk of colorectal cancer. Two potential mechanisms have been proposed for sertraline’s anti-cancer activity: 1. Sertraline interferes with the reciprocal repressive feedback loop between TCTP and the tumour suppressor P53 [31] by preventing the binding of TCTP to MDM2 and consequently the destabilisation of P53 caused by TCTP [29]. 2. Sertraline has been shown to inhibit translation initiation at the level of eIF4F complex formation, by an increase in eIF2α phosphorylation and by directly interfering with the mTORC1 pathway [86]. We have established that TCTP mRNA translation is subject to positive regulation through eIF4E [87] and the mTORC1 pathway [52], as well as to negative regulation by PKR and through eIF2α phosphorylation [21, 88]. It is therefore very likely that sertraline reduces TCTP levels also by inhibiting translation initiation. As mentioned before, other anti-apoptotic proteins are also translationally regulated through the mTOR pathway and therefore may be inhibited by sertraline; this has already been shown for Bcl-2 in CRC cells [83]. All these studies indicate that it would be worthwhile to further explore – and exploit - the anti-cancer activity of sertraline, alone or in combination with 5-FU or oxaliplatin in CRC.

### Cytoprotective role of TCTP in cancer

In order to demonstrate the functional importance of increased TCTP expression in response to 5-FU and oxaliplatin, we also asked whether increased TCTP levels are able to protect HCT116 cells against the cytotoxic effects of these drugs. Using cytotoxicity assays, we tested the effect of TCTP-knockdown on the sensitivity of these cells to 5-FU and oxaliplatin, and showed that it is indeed increased after TCTP-knockdown (Fig. 5). Consistent with this, TCTP overexpressing partially protected cells against 5-FU and oxaliplatin (Additional file 1). The cytoprotective effect of TCTP in cancer cells has been demonstrated for various cell stress conditions (reviewed in [11, 12, 14]). The involvement of TCTP (also named fortilin) in the protection of cancer cells against anti-cancer drugs has been reported in other settings as well, e.g. for etoposide in HeLa cells and in U2OS cells [89], and for 5-FU in U2OS cells [90].

It is therefore very likely that TCTP is involved in the development of anti-cancer drug resistance, a widespread problem in cancer chemotherapy. Indeed, an early study on chemoresistant melanoma cells showed that, among other proteins, TCTP is overexpressed in these cells [65]. A more recent study demonstrated the overexpression of TCTP (also called HRF) in non-Hodgkin lymphomas, and it established a role for this protein in cell adhesion and drug resistance [91]. On the other hand, a recent detailed investigation on gene signatures for drug resistance in HCT116 cells has failed to detect TCTP (TPT1) as a gene potentially regulated under these conditions [92]. However, this study was based entirely on microarray data and is therefore unlikely to detect translationally controlled genes.

Apart from being involved in chemoresistance of cancer cells, TCTP has been shown to play a significant role in the later stages of tumour development, which encompass the cellular properties of epithelial to mesenchymal transition (EMT), migration, invasion and metastasis. This has been demonstrated for a range of cancer cells [11, 39, 41], and specifically for colon cancer [51, 59]. Our observation that average TCTP levels increase early in CRC, with less elevation from the adenocarcinoma to the metastatic stage (Fig. 1), is consistent with the report from others [59], showing that TCTP levels of metastatic samples are not much higher than those of colon adenomas. One has to keep in mind that there is a considerable variability in TCTP levels between individual patients, within each, the non-metastatic and the metastatic group of adenocarcinomas. A high TCTP status has been associated with a poor patient outcome for CRC [59] and for a range of other cancer types [31–34]. All these findings suggest that TCTP is a potential biomarker and a target for anti-tumour therapy, which has been proposed for various types of cancers [11, 40–42].

## Conclusions

Our results on the expression of TCTP early in development in CRC support the view that TCTP could be potentially explored as a biomarker in colon cancer, to assist in the detection of early tumour growth and to identify patients with a high TCTP status. Indeed another study has provided initial results on increased serum TCTP levels in colon cancer patients [59]. The main conclusion from our results on colon cancer cells is that TCTP is very likely involved in the development of anti-drug resistance against 5-FU and oxaliplatin, the most widely used chemotherapeutic agents in CRC. It is therefore worthwhile to explore its suitability as a biomarker for chemoresistance and as an anti-cancer target in chemoresistant tumours. Our finding that up-regulation of TCTP in response to 5-FU and oxaliplatin is regulated through the mTORC1 pathway would suggest that combinatorial treatment with 5-FU/oxaliplatin, together with mTOR inhibitors, could be a suitable approach.

## Additional file

Additional file 1: TCTP overexpression partially protects mammalian cells against the cytotoxic effects of 5-FU and oxaliplatin. We utilised a previously established model cell line that stably overexpresses TCTP [26]. These are bovine mammary epithelial cells, where one line harbours a TCTP-overexpressing plasmid (white bars), and the control cell line the empty vector (black bars). The cells were cultured in DMEM supplemented with 10% FCS, 50 mg/ml hygromycin B and 5 mg/ml insulin. Cells were subjected to treatment with the indicated concentrations of 5-FU or oxaliplatin. Cell growth was monitored in real-time using the xCELLigence RTCA System (left graphs), or using the MTS endpoint assay for cytotoxicity (right graphs). Cell numbers were plotted as relative cell index (CI) after 72 h of drug treatment (left graphs), or as relative cell viability for the MTS assay after 48 h of treatment (right graphs), against the indicated concentrations of 5-FU (top graphs) or oxaliplatin (bottom graphs). (PPTX 283 kb)

## Abbreviations

5’-TOP: 5’-Terminal oligopyrimidine tract
5-FU: 5-fluorouracil
Akt: Synonym for protein kinase B (PKB)
CEA: Carcino-embryonic antigen
CI: Cell Index (indication of cell growth; scoring of the RTCA system)
CRC: Colorectal cancer
DAB: 4-Dimethylaminoazobenzene
DTT: Dithiothreitol
EDTA: Ethylenediamine tetraacetic acid
EMT: Epithelial to mesenchymal transition
FCS: Foetal calf serum
GAPDH: Glyceraldehyde phosphate dehydrogenase
HE: Haematoxylin-eosin (staining)
HRP: Horseradish peroxidase
IC_50_: Inhibitory concentration resulting in 50% cell death
M-MLV: Moloney murine leukemia virus
mTORC1: Mechanistic target of rapamycin complex 1
MTS assay: Cell proliferation assay (see Methods)
p70-S6 kinase: Ribosomal protein S6 kinase, molecular mass: 70 kDa
PBS: Phosphate-buffered saline
PI3-kinase: Phosphoinositol 3-phosphate kinase
PVDF: Polyvinylidene difluoride
rpm: Revolutions per minute
rpS6: Ribosomal protein S6
RTCA System: real-time cell analysis system
RT-qPCR: Reverse transcription quantitative PCR
SD: Standard deviation
SEM: Standard error of the mean
siRNA: Small inhibitory ribonucleic acid
SSRI: selective serotonin reuptake inhibitor
TCTP: Translationally controlled tumour protein (gene symbol: TPT1); also called HRF histamine releasing factor, or fortilin
TNM stage IV: Stage four of the international tumour staging system; T – tumour size N – number of lymph nodes involved, M – number of metastases
