# Supplementary figures and images for: Translationally controlled tumour protein TCTP is induced early in human colorectal tumours and contributes to the resistance of HCT116 colon cancer cells to 5-FU and oxaliplatin
Source: Cell Commun Signal. 2017 Feb 1;15:9. doi: 10.1186/s12964-017-0164-3 (PMC5286767; doi:10.1186/s12964-017-0164-3)

## Slide 1
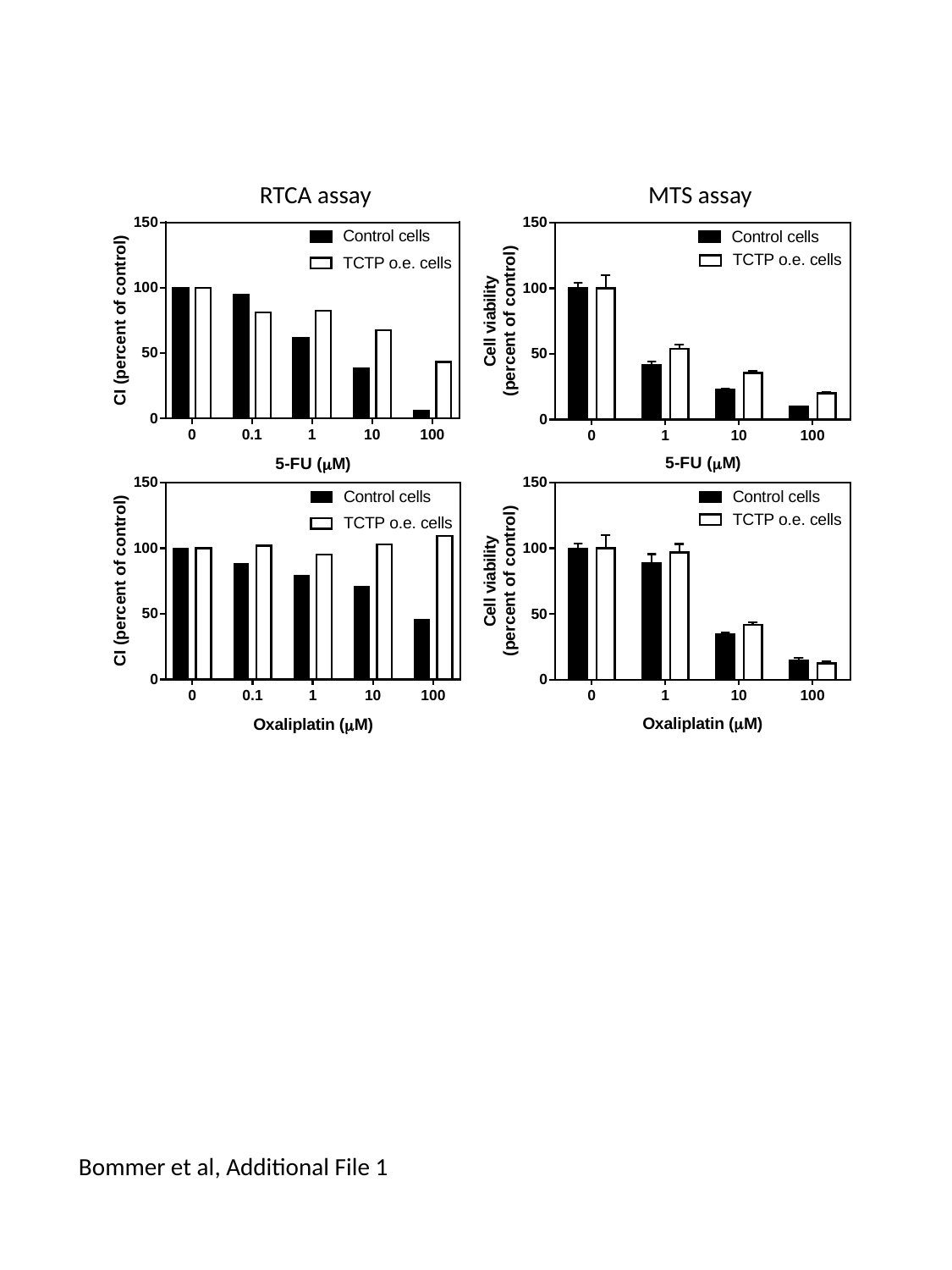

RTCA assay
MTS assay
Bommer et al, Additional File 1

Supplement: Additional file 1: — TCTP overexpression partially protects mammalian cells against the cytotoxic effects of 5-FU and oxaliplatin. We utilised a previously established model cell line that stably overexpresses TCTP [26]. These are bovine mammary epithelial cells, where one line harbours a TCTP-overexpressing plasmid (white bars), and the control cell line the empty vector (black bars). The cells were cultured in DMEM supplemented with 10% FCS, 50 mg/ml hygromycin B and 5 mg/ml insulin. Cells were subjected to treatment with the indicated concentrations of 5-FU or oxaliplatin. Cell growth was monitored in real-time using the xCELLigence RTCA System (left graphs), or using the MTS endpoint assay for cytotoxicity (right graphs). Cell numbers were plotted as relative cell index (CI) after 72 h of drug treatment (left graphs), or as relative cell viability for the MTS assay after 48 h of treatment (right graphs), against the indicated concentrations of 5-FU (top graphs) or oxaliplatin (bottom graphs). (PPTX 283 kb) [file 12964_2017_164_MOESM1_ESM.pptx]
